# Structure-based drug designing and immunoinformatics approach for SARS-CoV-2

**DOI:** 10.1126/sciadv.abb8097

**Published:** 2020-07-10

**Authors:** Pritam Kumar Panda, Murugan Natarajan Arul, Paritosh Patel, Suresh K. Verma, Wei Luo, Horst-Günter Rubahn, Yogendra Kumar Mishra, Mrutyunjay Suar, Rajeev Ahuja

**Affiliations:** 1 Condensed Matter Theory Group, Materials Theory Division, Department of Physics and Astronomy, Uppsala University, Box 516, SE-751 20 Uppsala, Sweden.; 2 Department of Theoretical Chemistry and Biology, Royal Institute of Technology (KTH), AlbaNova University Center, 106 91 Stockholm, Sweden.; 3 School of Biotechnology, KIIT University, Bhubaneswar 751024, India.; 4 Syddansk Universitet, Alsion 2, DK-6400 Sønderborg, Denmark.; 5 Mads Clausen Institute, NanoSYD, University of Southern Denmark, Alsion 2, DK-6400 Sønderborg, Denmark.; 6 Applied Materials Physics, Department of Materials Science and Engineering, Royal Institute of Technology (KTH), SE-100 44 Stockholm, Sweden.

## Abstract

The prevalence of respiratory illness caused by the novel SARS-CoV-2 virus associated with multiple organ failures is spreading rapidly because of its contagious human-to-human transmission and inadequate globalhealth care systems. Pharmaceutical repurposing, an effective drug development technique using existing drugs, could shorten development time and reduce costs compared to those of de novo drug discovery. We carried out virtual screening of antiviral compounds targeting the spike glycoprotein (S), main protease (M^pro^), and the SARS-CoV-2 receptor binding domain (RBD)–angiotensin-converting enzyme 2 (ACE2) complex of SARS-CoV-2. PC786, an antiviral polymerase inhibitor, showed enhanced binding affinity to all the targets. Furthermore, the postfusion conformation of the trimeric S protein RBD with ACE2 revealed conformational changes associated with PC786 drug binding. Exploiting immunoinformatics to identify T cell and B cell epitopes could guide future experimental studies with a higher probability of discovering appropriate vaccine candidates with fewer experiments and higher reliability.

## Teaser

We report development of in-silico approaches for the identification of anti-coronaviral drugs against SARS-CoV-2.

## INTRODUCTION

**Fig. 1:**
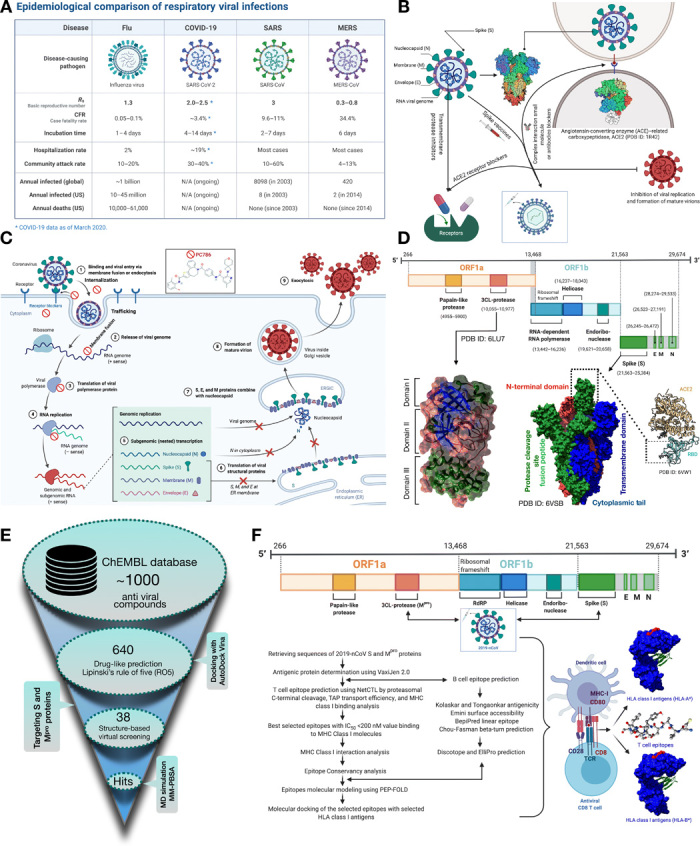
Repurposed therapeutics for SARS-CoV-2 with broad-spectrum antiviral activities. (**A**) Epidemiological comparison of the recent outbreak SARS-CoV-2 with previous respiratory viral infections. N/A, not applicable. (**B**) In silico proposed analyses targeting trimeric S protein, M^pro^, and trimeric S protein RBD-ACE2 complex. (**C**) Potential repurposed drug candidates for SARS-CoV-2 targeting viral entry mechanism. (**D**) Genomic organization of SARS-CoV-2 with structural domains representing M^pro^ and trimeric S protein (surface representation with the colors indicating secondary structure, i.e., blue, β strands; green, helix; brown, Coils for M^pro^) (green, chain A; blue, chain B; red, chain C for trimeric S protein). The RBD of trimeric S protein involved in the interaction with the human host ACE2 enzyme is shown. (**E**) Virtual library screening workflow discerning repurposed antiviral drugs targeting trimeric S and M^pro^ of SARS-CoV-2. (**F**) Approaches to predict potential vaccine candidates (T cell and B cell epitopes) for SARS-CoV-2. Schematic representation of the major histocompatibility I (MHC) class I displaying antigenic peptides to CD8 T cells. The surface representation of the human leukocyte antigen (HLA) MHC class I molecules bound to T cell epitopes (red color). IC_50_, median inhibitory concentration.

A new coronavirus disease previously known as 2019-nCoV (2019 novel coronavirus) but later known as SARS-CoV-2 (severe acute respiratory syndrome coronavirus 2) has recently emerged from China with a total of >4 million confirmed cases and > 300 thousand deaths worldwide (*1*). Similar to SARS-CoV, SARS-CoV-2 tends to transfer rapidly from human to human, distributed across multiple continents (*2*). Epidemiological studies help determine the health status of a nation and enable a better distribution of economic resources. An epidemiological data source of Respiratory Viral Infections from Yale and BioRender scientific team shows that COVID-19 (coronavirus disease 2019) has a high rate of hospitalization due to the high mortality rate as well as the declaration of SARS-CoV-2 as a pandemic by the World Health Organization. The community attack rate is way much higher in comparison to other respiratory viral infections, as shown in Fig. 1A. To mitigate this challenge, several researchers from all over the world try to develop or repurpose antiviral drugs through experimental and computational methods to diminish the fear of this pandemic outbreak.

The first reported genome sequencing of SARS-CoV-2 lead the researchers to determine a key target, the SARS-CoV-2 spike (S) glycoprotein for therapeutic and diagnostics (*3*). The S protein of SARS-CoV-2 [Protein Data Bank (PDB) ID: 6VSB] contains a receptor binding domain (RBD), which interacts with the peptidase domain of the angiotensin-converting enzyme 2 (ACE2) (*4*), thus mediates receptor recognition and membrane fusion (*5*). Research into finding appropriate drug compounds targeting the S protein RBD-ACE2 complex facilitated through virtual screening of drug compounds computationally is in pursuit to ensure potential treatments (*6*). Another vital enzyme, i.e., SARS-CoV-2 main protease (M^pro^) (PDB ID: 6 LU7) (*7*), that intercedes viral replication and transcription together with the absence of closely related homolog proteins in humans can be a potential target for therapeutics development. In the mission to stop the outbreak, developing medical countermeasures using molecular modeling, virtual screening of drug candidates along with receptor-drug molecular dynamics (MD) simulation can facilitate the ease of finding antiviral drugs for SARS-CoV-2. The plan to reuse old drugs introduced for past outbreaks, e.g., MERS-CoV, SARS-CoV, Ebola, and HIV, could, therefore, accelerate the discovery process (*8*).

We have devised a similar strategy using in silico approaches that include (i) inhibition of the S protein, M^pro^, and RBD-ACE2 complex interaction using small molecules/antiviral drugs and (ii) immunoinformatics approach for designing specific epitopes of major histocompatibility complex (MHC) class I antigens for adaptive immunity using S protein and M^pro^ (Fig. 1B). Both the computational strategies could pave a path for the experimentalists and pharmaceutics companies to design drugs and vaccines for this SARS-CoV-2 in a short period. Repurposing of potential drug candidates having a broad-spectrum antiviral activity targeting the viral entry mechanism could be beneficial for clinical use. The current study deals with a similar strategy to find potential drug or vaccine candidates suitable for possible experimental studies targeting the infection pathway of SARS-CoV-2.

The key mechanism underlying the SARS-CoV-2 replication and maturation of the virion mediated through host cell attachment is illustrated in Fig. 1C. The interaction of spike protein (RBD) initiates the coronavirus attachment to the host cell to the host receptor through membrane fusion and endocytosis (Fig. 1C). Followed by the receptor binding, the release of the viral genome is accomplished by acid-dependent proteolytic cleavage of the S protein by a protease enzyme. The translation of the viral polymerase protein starts after using a RNA pseudoknot and a slippery sequence (5′-UUUAAAC-3′) that causes ribosomal frameshifting. The assembly of the viral replicase complexes follows the translation and assembly by producing genomic and subgenomic RNAs. The genomic and subgenomic RNAs were produced through negative-strand intermediates and from which the subgenomic RNAs go through nested transcription. The spike (S), membrane (M), and envelope (E) proteins then undergo translation or assembly through insertion to endoplasmic reticulum (ER) that moves along a secretory pathway into the ER-Golgi intermediate compartment (ERGIC). There, viral genomes encapsidated by a (nucleocapsid) N protein bud into membranes of the ERGIC containing viral structural proteins, forming mature virions. Upon assembly, vesicles transport virions to the cell surface and induce exocytosis (*9*).

A variable number (6 to 11) of open reading frames (ORFs) are included in the coronavirus genome, which has a size from about 26,000 to 32,000 bases. The first ORF codes 16 nonstructural proteins, covering almost 67% of the entire genome. The other ORFs include accessory proteins and structural proteins. The 5′ gene contains more than two-thirds of the orf1ab encoding orf1ab polyproteins. The 3′ gene comprises structural-protein encoding (S), envelope (E), membrane (M), and nucleocapsid (N) proteins (*10*). (Fig. 1D) The cryo–electron microscopy structure of SARS-CoV-2 trimeric spike (S) glycoprotein (PDB ID: 6VSB) determined by Wrapp *et al.* (*3*) is considered to be a key target in therapeutics and diagnostics of this pandemic coronavirus spread. The spike protein is in a state of trimeric form with three RBDs and in metastable prefusion conformation that undergoes structural rearrangements to fuse the viral membrane with the host human cell membrane. The RBDs undergo hinge-like conformational movements when attached with the ACE2 host receptor. The crystal structure of SARS-CoV-2 M^pro^ (PDB ID: 6 LU7) consists of three domains, i.e., domains I and II have a chymotrypsin-like and two–β-barrel fold conformations and domain III consists of five helices that adopt a globular structure (*11*). The coronavirus M^pro^ enzyme is essential for proteolytic maturation of the virus. It is a promising target for the discovery of small-molecule drugs that would inhibit cleavage of the viral polyprotein and prevent the spread of the infection.

There are currently no scientifically appropriate vaccinations or unique antiviral therapy for the prevention or treatment of COVID-19. The α-interferon mixture and anti–HIV lopinavir/ritonavir (Kaletra) (*12*) medications have been used, but there is still a minimal curative benefit, and toxic side effects can occur (*13*). Remdesivir is also being explored for the treatment of COVID-19, a broad-spectrum antiviral developed by Gilead Sciences Inc., but validation from clinical studies is needed to demonstrate its effectiveness (*14*). On the basis of the recent research report, we computationally screened 640 antiviral compounds from the ChEMBL (*15*) database against the S protein and M^pro^ using AutoDock Vina (Fig. 1E) (*16*). We have used UCSF (University of California at San Francisco) Chimera (*17*) and Discovery Studio Visualizer (*18*) for the postdocking analyses. An antiviral polymerase inhibitor PC786 bearing ChEMBL ID 4291143 proved to be the best among all the antiviral drugs against both the target receptors. Apart from PC786, several other antiviral drugs, i.e., lorecivivint, tegavivint, and dolutegravir, also have better binding affinities toward S and M^pro^.

Furthermore, we have also compared the binding affinities of the U.S. Food and Drug Administration (FDA)–approved drugs against these two target receptors, along with the RBD-ACE2 complex. The screened drugs were proven more effective than the FDA-approved medications in terms of binding affinities. We also combined the MD simulation with a virtual screening strategy to validate the binding strength of the PC786 drug in comparison to the FDA-approved drugs, i.e., zanamivir and lopinavir. Moreover, upon interaction with the PC786 drug, the trimeric S protein RBD in complex with ACE2 seems to change its conformation. ACE2 directly interacts with the RBD in a close conformation with high binding affinity in its native state, whereas, in the case of the PC786 drug bound to trimeric S protein, the RBD seems to interact with ACE2 in an open conformation.

In addition to the drug screening method, the application of immunoinformatics using a bioinformatics approach to the design of different vaccine candidates for SARS-CoV-2 serves as an alternative and promising approach (Fig. 1F). Active counteractions to the recent appearance and rapid expansion of the SARS-CoV-2 entail the creation of data and resources to identify and track its spread and immune response. As of 27 January 2020, the Immune Epitope Database and Analysis Resource (IEDB) curated 581 linear and 81 discontinuous B cell epitopes along with 320 T cell epitopes for SARS-CoV-2 (*19*). Similarly, we have also predicted T cell and B cell epitopes using structural sequences of S and M^pro^ proteins of SARS-CoV-2 using IEDB resources (*20*). Independent detection of the epitopes using the vital proteins illustrates the high likelihood of identifying vaccine candidates for the immune responses to SARS-CoV-2. These forecasts will promote the successful design of vaccinations against this high-priority virus.

## RESULTS

### Structure-based drug design approach: Screening of ChEMBL antiviral compounds

**Fig. 2:**
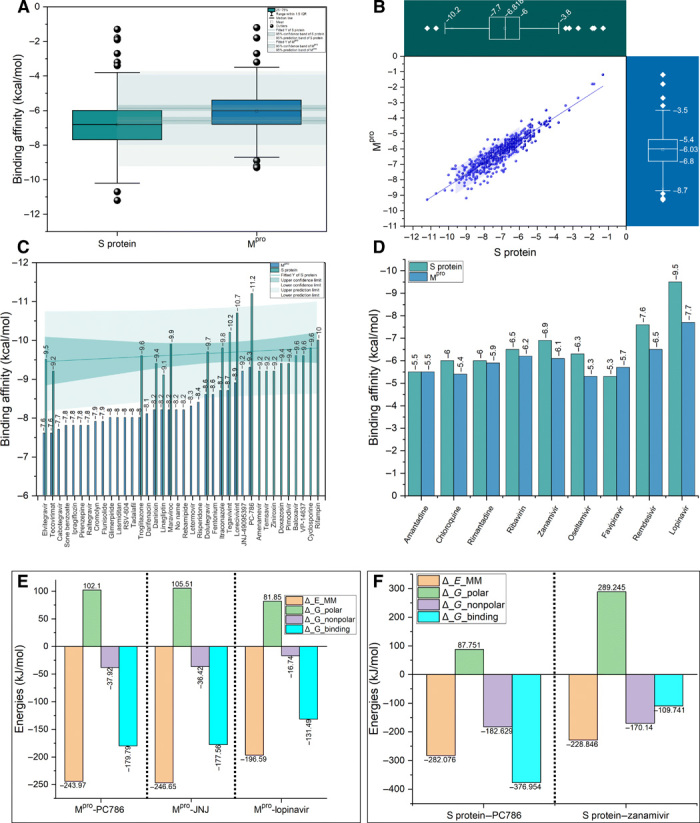
Virtual screening of potential antiviral drugs against S and M^pro^ proteins of SARS-CoV-2. (**A**) Box plot with normal distribution with two different groups of the same size (*n* = 640) showing the antiviral compounds binding affinities in kilocalorie per mole for trimeric S protein (green) and M^pro^ (blue) with optimal means and 95% confidence intervals (CI). The outliers were shown in black spheres. IQR, interquartile range. (**B**) Two-dimensional (2D) scatter plot (blue color) showing the distribution of binding affinity scores in kilocalorie per mole for all the virtually screened *n* = 640 antiviral drugs against both trimeric S protein and M^pro^. The two box and whisker plots (green color for S protein and blue color for M^pro^) shows the distribution of binding affinity scores (kcal/mol) with median (*m* = −6.81 and –6.03 kcal/mol) for S protein and M^pro^, respectively. A horizontal box chart represents the S protein scatter data. A vertical box chart represents the M^pro^ scatter data. (**C**) Bar chart showing the binding affinity scores (kcal/mol) for the selected antiviral compounds ranging from −7.5 to −12.0 kcal/mol) with 95% CI (green shade). The maximum binding affinity (high negative scores indicates maximum binding affinity) is shown. (**D**) Bar chart showing binding affinities scores in kilocalorie per mole for selected approved drugs. (**E** and **F**) Free energy terms obtained from MM-PBSA calculations relative to two selected drugs from virtual screening for M^pro^ and one for S protein in comparison to approved drugs.

We have screened the ChEMBL database for antiviral drugs that have passed the Lipinski’s rule of five (RO5) for drug-likeliness (Fig. 1E). The structure-based drug design approach was taken into consideration using both the S protein and the M^pro^ of SARS-CoV-2. The antiviral drugs obtained from the ChEMBL database have been listed in data file S1. High-throughput virtual screening of the antiviral drugs using a molecular docking approach resulted in a broad range of binding affinity toward both the receptors (Fig. 2, A and B), typically ranging from −1.5 to −11.5 kcal/mol. Among all the antiviral drugs, PC786 has been considered to have the highest binding affinity toward both the target receptors (Fig. 2C). The PC786 drug bearing ChEMBL ID 4291143 has already passed phase 1 and 2 clinical trials. It is a non-nucleotide inhibitor of respiratory syncytial virus (RSV) polymerase that inhibits replication of both the A and B subtypes of acute RSV, thus interrupting the spread of infection within the respiratory tract. The inhalation administration of PC786 leads to high local airway concentration at a viral replication site with low systemic toxicity and, therefore, a low risk of systemic side effects. Via phase 1 research, PC786 shows excellent safety and tolerability. Preclinical and clinical pharmacokinetic data show low systemic concentrations with prolonged lung retention (*21*). The molecular docking analysis also revealed that PC786 has the best binding affinity of −11.3 kcal/mol with S protein (fig. S1A) and −9.3 kcal/mol with the M^pro^ of SARS-CoV-2 (fig. S1K and Fig. 2C).

The findings are the basis for the repurpose of the approved/investigational small molecules against SARS-CoV-2 infection. We have also compared the relative binding affinities of screened antiviral compounds with the FDA-approved drugs using the two target receptors. The results showed superior binding affinities of computationally screened viral compounds, i.e., PC786, when compared to those drugs under clinical trials (except the drug lopinavir) (Fig. 2D). Lopinavir has shown the highest binding affinity toward M^pro^ with the binding affinity of −9.5 kcal/mol, which is comparatively less than the PC786 drug. Similarly, remdesivir and zanamivir have shown high binding affinities toward trimeric S protein, i.e., −7.6 and −6.9 kcal/mol. It is known that remdesivir targets only a highly conserved RNA-dependent RNA polymerase in diverse RNA viruses, providing a basis for designing broad-spectrum antiviral drugs based on nucleotide analogs (*14*). Therefore, for further validation and comparison of the FDA-approved antiviral drugs with the best-screened drugs, we used MD simulation along with molecular mechanics Poisson-Boltzmann surface area (MM-PBSA) calculations (fig. S1S and Fig. 2, E and F). The findings from the MD and MM-PBSA calculations are further explored in the MD section.

### Molecular docking analyses of screened drugs against S and M^pro^

**Fig. 3:**
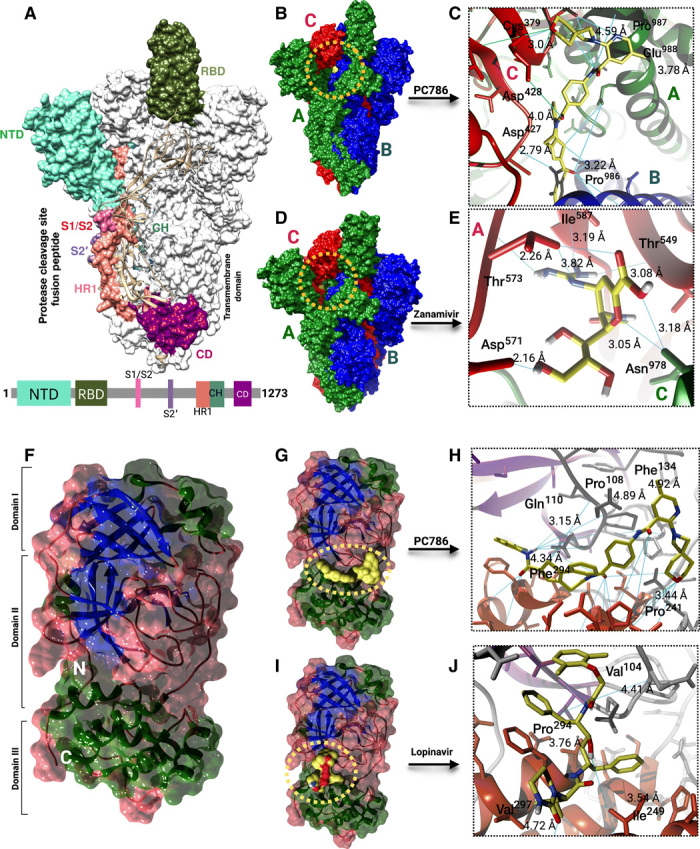
Antiviral drugs binding modes for S and M^pro^. (**A**) Surface representation of the trimeric S protein of SARS-CoV-2 with structural domains. Schematic of S protein primary structure colored by the domain (below). NTD, N-terminal domain; RBD, receptor binding domain; S1/S2, S2’, protease cleavage site; HR1, heptad repeat 1; CH, central helix; CD, connector domain. (**B** and **D**) PC786 and zanamivir binding mode (yellow) to S protein (colors indicating chains). (**C** and **E**) Close-up view of PC786 (yellow surface) and zanamivir (yellow sticks) binding to S protein chains (ribbons). Sky blue lines indicate hydrogen bonds. (**F**) Surface representation of the SARS-CoV-2 M^pro^ structural domains. The structure is represented by its secondary structure components **(**blue, β strands; green, helix; brown, coils). (**G** and **I**) PC786 and lopinavir binding mode (yellow) to M^pro^. (**H** and **J**) Close-up view of PC786 (yellow surface) and lopinavir (yellow surface) binding to M^pro^ (ribbons). Sky blue lines indicate hydrogen bonds.

Our in silico strategy enables us to design and screen the small molecules targeting the trimeric S protein that contains key structural domains (Fig. 3A). Specifically, RBD plays a pivotal role in viral-host attachment (*3*). These specific structural domains can be targeted with small molecules to disrupt the viral attachment to the host proteins. From the postdocking interaction analysis, PC786 has shown most favorable binding affinity toward RBD of all the chains in trimeric S protein. PC786 also targets the junction of the heptad repeat 1 (HR1)/ central helix (CH) domain. The specificity of PC786 binding to RBD of trimeric S protein could be of potential interest for experimentalists or clinicians for further validation. PC786 drug interaction with the trimeric S protein showed conventional carbon-hydrogen bonds with Gly^413^ of B chain and Asp^427^ and Cys^379^ of C chain, halogenic bond with Pro^986^ B chain, and alkyl bonds with Leu^752^ and Pro^987^ (Fig. 3, B and C, and fig. S2A). The halogen bond with Pro^986^ indicates that there is a net attractive interaction between an electrophilic region associated with a fluorine atom and a nucleophilic region Pro^986^. π-anion bond with Glu^988^ also has been observed for the same. Apart from PC786, lorecivivint and tegavivint also yielded good binding affinity of −10.7 kcal/mol, −10.2 kcal/mol in the case of trimeric S protein (fig. S1, B and C).

Lorecivivint is a phase 2 clinical trial anti-inflammatory drug that inhibits or even reverses the progression of osteoarthritis. Tegavivint is an antineoplastic drug that inhibits the Wnt/β-catenin pathway with potential antineoplastic activity. Among the FDA-approved drugs, zanamivir turned out to be the second best (next to remdesivir) having a binding affinity of −6.9 kcal/mol with S protein (fig. S1D). Zanamivir showed conventional hydrogen bodings with Asn^978^ and Val^976^ of A chain, and the rest of the key residues involved were from the C chain of trimeric S protein (Fig. 3, D and E, and fig. S2D). In the case of lorecivivint, there are many conventional hydrogen bonds with interacting with the A chain of the S protein, i.e., the O atom and N atom of the lorecivivint forms bond with Arg^1014^ and Arg^1019^, respectively (fig. S2B). In the case of tegavivint, many alkyl bonds and an unfavorable donor-donor bond with Arg^1014^ of A chain seem to be formed during the interaction with the trimeric S protein of SARS-CoV-2 (fig. S2C). All the bonding patterns mentioned above that have been interacted with the spike protein are in the vicinity of the active site pockets, as illustrated in fig. S1I and data file S2. The target receptor hydrogen bonding and solvent accessible surfaces were depicted in fig. S1 (E to H).

The SARS-CoV-2 M^pro^ protein tends to show high binding affinities with PC786 with a binding affinity of −9.3 kcal/mol. JNJ-449095397 (JNJ), which is an inhibitor for chronic obstructive pulmonary disease, also has high the binding affinity of −9.2 kcal/mol with the M^pro^ of SARS-CoV-2 (fig. S1L). The interaction analysis of the best-screened drugs with M^pro^ revealed that the drugs mostly binds to junction of domains II and III that have two–β-barrel fold conformations and five helices (Fig. 3F).

PC786 showed conventional hydrogen bonds with Gln^110^ and many hydrophobic interactions with Phe^134^, Pro^108^, Ile^249^, Val^202^, Pro^203^, and Phe^294^ (Fig. 3, G and H), whereas JNJ showed conventional hydrogen bonds with Lys^137^, Thr^199^, Tyr^239^, Asn^277^, and Met^276^ (figs. S1, K and L, and S2, E and F**)**. In addition, lorecivivint also showed a better binding affinity of −8.9 kcal/mol that has a high binding affinity than FDA-approved drug lopinavir (figs. S1M and S2G) with conventional hydrogen and halogen bonds. In the case of FDA-approved drug lopinavir, Pro^293^, Val^297^, Val^104^, and Ile^206^ form hydrophobic interactions with the drug molecule along with two π-σ bonds associated with Ile^249^ and Phe^294^ (Fig. 3, I and J, and figs. S1N and S2H). All the interacted residues in the M^pro^ protein were in the active site region, as shown in fig. S1J and data file S2. The target receptor hydrogen bonding and solvent accessible surfaces were depicted in fig. S1 (O to R). The final screened best antiviral and FDA-approved compounds with virtual screening binding affinity scores have been provided in data file S1. The therapeutic description of best selected antiviral drugs was also described in data file S1.

### MD simulation with free energy (MM-PBSA) calculations

**Fig. 4:**
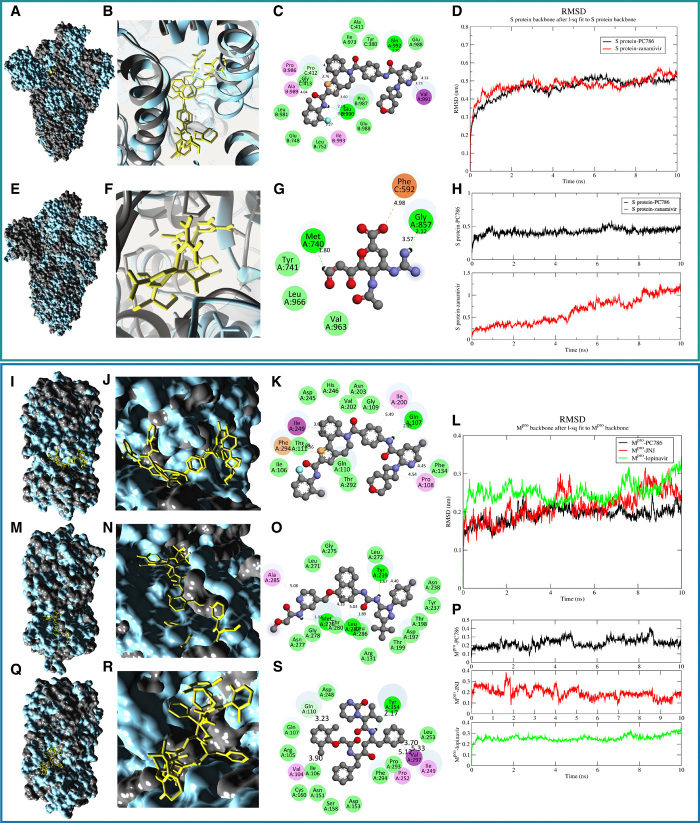
MD simulation of the proposed antiviral drugs bound to trimeric S protein and M^pro^. The green panel shows the MD simulation results for S protein drug conjugates, and the blue panel represents the M^pro^ protein drug conjugates. (**A** and **E**) Surface representation of the trimeric S protein from 10-ns simulation (gray, initial configuration of trimeric S protein at 0 ns; cyan, 10-ns simulated structure) (**B** and **F**) Conformations of the antiviral drugs with S protein, i.e., PC786 and zanamivir (yellow sticks), respectively, after 10-ns simulation. (**C** and **G**) 2D representation of the drug interaction (key residues involved in the interaction mechanism. Green circles representing conventional hydrogen bonds; pink, alkyl bonds; violet, π-σ bonds; orange, pi-anion bonds; yellow; pi-sulfur bonds). (**D**) RMSD plot for the trimeric S protein during the 10-ns simulation. (**H**) RMSD plot of the antiviral drugs. (**I**, **M**, and **Q**) Surface representation of the M^pro^ from 10-ns simulation (gray, initial configuration of M^pro^ protein at 0 ns; cyan, 10-ns simulated structure). (**J**, **N**, and **R**) Conformations of the antiviral drugs with M^pro^, i.e., PC786, JNJ, and lopinavir (yellow sticks), respectively, after 10-ns simulation. (**K**, **O**, and **S**) 2D representation of the drug interaction [critical residues involved in the interaction mechanism; refer to (C) and (F) for legends]. (**L**) RMSD plot for M^pro^ proteins conjugated with drugs during 10-ns simulation. (**P**) RMSD plot of the antiviral drugs.

To validate the intrinsic atomic interaction and binding conformation of the best-screened antiviral drugs, we have used all atom-based MD simulation using GROMACS v.2019.2 (*22*) for 10 ns for the protein-drug conjugates. Figure 4 (A to S) illustrates the results from MD calculations, i.e., conformations of the proteins bound to the antiviral drugs and the molecular interactions antiviral drugs. We have taken PC786 and zanamivir with S protein (Fig. 4, A to H) and PC786, JNJ, and lopinavir with M^pro^ (Fig. 4, I to S). The clusters of the 10-ns simulated structures for S protein and M^pro^ conjugated with the drugs mentioned above were illustrated in figs. S3 (A and B) and S4 (A to C), respectively. We have taken the FDA-approved drugs zanamivir and lopinavir to compare with the virtually screened drugs against S protein and M^pro^, respectively, as the binding affinities of the respective compounds is higher toward respective target receptors. We have compared the 10-ns simulated structure with the initial configuration (0 ns) using the structure comparison tool (MatchMaker) from UCSF Chimera to align the pair of protein chains that uses BLOSUM62 matrix and Needleman-Wunsch algorithm. Figure 4 (A and E) represents the overlap of 0- to 10-ns simulated structure of S protein bound to PC786 and zanamivir, respectively. Similarly, we have compared the simulated structure of M^pro^ bound to PC786, JNJ, and lopinavir that are illustrated in Fig. 4 (I, M, and Q), respectively.

The 10-ns MD simulation revealed that the trimeric S protein seems to be stable that showed root mean square deviation (RMSD) in the range of ~0.2 to ~0.5 nm in both the drug conjugates, i.e., PC786–trimeric S protein and zanamivir–trimeric S protein (Fig. 4D), respectively. The ligand (drug molecules) bound to trimeric S protein seems to fluctuate during the 10-ns simulation that varied in the RMSD range of ~0.15 to ~1.3 nm as shown in Fig. 4H for both systems, respectively. PC786 showed a slight deviation in terms of its structural conformation (Fig. 4B) from which the stability of the drug can be delineated, whereas notable difference can be observed in the case of zanamivir (Fig. 4F). Similarly, root mean square fluctuation (RMSF) analysis also showed high fluctuations in zanamivir compared to PC786 (fig. S3, E and F). The compactness of the S protein receptor upon binding with zanamivir has shown a substantial decrease in radius of gyration (Rg) values (fig. S3G). PC786 seems to retain the native bonding patterns (at 0 ns), whereas zanamivir showed substantial deviation in bonding patterns (Fig. 4, C and G), respectively. Moreover, the total interaction energies calculated from the average short-range Coulombic interaction energy and the short-range Lennard-Jones energy are higher in the case of zanamivir that shows a less binding affinity toward the target receptor (likely to deviate from its original orientation) (fig. S3, C and D).

Furthermore, to estimate the interaction free energies of the biomolecular interaction, the complex systems have been subjected to MM-PBSA calculations (*23*). The combination of MD with MM-PBSA incorporates conformational fluctuations and entropic contributions to the binding energy. The binding energy decomposition analysis divulged into various free energies associated with the ligand upon binding to the protein, e.g., Δ*E*_MM_, Δ*G*_polar_, and Δ*G*_nonpolar_, was calculated separately and later combined to predict the total energies of the individual components. The net contribution energy of the residues involved in the interaction to the drug molecules has been depicted in fig. S3H and data file S3. The energy components such as Δ*E*_MM_, Δ*G*_polar_, and Δ*G*_nonpolar_ were calculated for 10 ns extracted at each 10-ps interval from the production trajectories. The binding energy Δ*G*_binding_ calculated from MM-PBSA calculation is higher in magnitude in the case of PC786 (−376.95 kJ/mol) as compared to zanamivir (−109.74 kJ/mol) (Fig. 2F).

A similar approach has been undertaken for M^pro^ as well, where we compared PC785 and JNJ with FDA-approved drug lopinavir as it showed high binding affinity. The RMSD of the M^pro^ protein tends to deviate more in the case of lopinavir as compared to PC786 and JNJ (Fig. 4L). The ligand RMSD of PC786, JNJ, and lopinavir showed comparatively similar behavior in terms of RMSD (Fig. 4P) and can be visualized in Fig. 4 (J, N, and R), respectively. However, the RMSF of lopinavir tends to have more fluctuation as compared to PC786 and JNJ (fig. S4, F and G), whereas the compactness of the proteins remains the same in all three cases (fig. S4H). Figure 4 (K, O, and S) shows the two-dimensional (2D) interaction plots of PC786, JNJ, and lopinavir interacted with M^pro^ after 10-ns simulation.

The total interaction energy of lopinavir is very high compared to PC786 and JNJ (fig. S4, D and E). The binding energies Δ*G*_binding_ of PC786 and JNJ (−179.79 and −177.56 kJ/mol) are higher in magnitude as compared to lopinavir (−131.49 kJ/mol) (Fig. 2E). The net contribution energy of the residues involved in the interaction to the drug molecules has been depicted in fig. S4I and data file S3. The contributions from van der Waals interactions are −247.146 and −233.90 kJ/mol to the total binding free energies of PC786 with S protein and M^pro^, respectively, suggesting that the complexation process is driven by hydrophobic interactions. If we compare the known antivirals, the contributions from van der Waals interactions are −92.82 kJ/mol in the case of zanamivir with S protein, whereas lopinavir has a van der Waals contribution of −188.88 kJ/mol with M^pro^. In both the cases, i.e., S and M^pro^, PC786 binding free energy and van der Waals contributions are higher than all the other known antiviral compounds that determine its specificity. Thus, the binding free energy calculations confirmed the favorable binding of antiviral drugs with the SARS-CoV-2 S and M^pro^ proteins and demonstrate the use of computational screening and free energy calculations on compounds from open-source chemical space toward successful research in drug design.

### Conformational changes of trimeric S protein RBD-ACE2 complex upon interaction with antiviral drugs

**Fig. 5:**
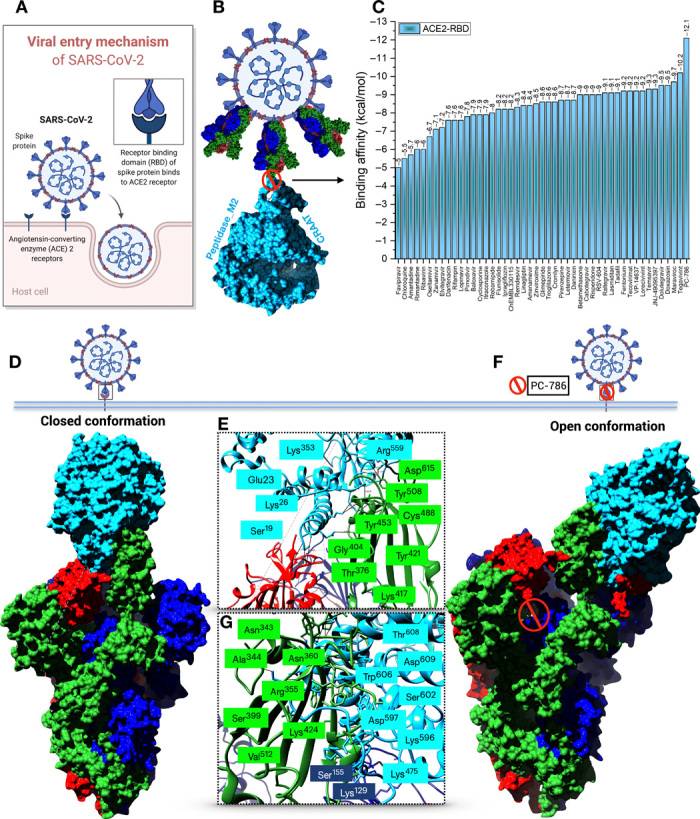
Structural basis of the RBD-ACE2 complex protein-protein interaction. (**A**) Schematic illustration of the viral entry mechanism of SARS-CoV-2. (**B**) Structural representation of the trimeric S protein RBD interaction inhibition with ACE2 by repurposed antiviral drugs. (**C**) Bar plot depicting binding affinities (kcal/mol) of selected antiviral drugs from virtual screening to RBD-ACE2 complex. (**D**) ACE2 binding to trimeric S protein RBD in a closed conformation. (**E** and **G**) Key residues involved in the interaction mechanism. Blue-colored residues are from ACE2 enzyme and green-shaded residues from trimeric S protein (**F**) Open conformation of antiviral drug (PC786) conjugate RBD-ACE2 complex.

The trimeric S protein RBD mediates receptor recognition and membrane fusion upon interaction with the ACE2 enzyme (Fig. 5A). The trimeric S protein S1 subunit that contains the RBD directly binds to the host receptor ACE2, which may exploit host infection. The S protein S2 subunit is mainly responsible for membrane fusion that is exposed and is cleaved by the ACE2 protease domain that is critical for viral infection. Initially, we have performed an ensemble docking of best-screened drugs obtained from virtual high-throughput screening (Fig. 5B) with the complex structure. The ensemble docking provides us similar results obtained using single target receptors, i.e., S protein and M^pro^. Again, PC786 was proven to be having a higher binding affinity toward the complex as well (−12.1 kcal/mol) (Fig. 5C). Furthermore, we have checked the binding affinity of PC786 with another configuration of the complex of RBD-ACE2 involving only the RBD (chain A) of trimeric S protein interacted with ACE2 (fig. S5, A and B). The binding affinity of −12.1 kcal/mol was obtained majorly involving residues of ACE2 protein. His^374^ and His^505^ of ACE2 form conventional hydrogen bonding with the PC786 drug (fig. S5C). The full trimeric S protein RBD-ACE2 complex conjugated with PC786 drug results in forming conventional hydrogen bonding with Cys^379^ with the binding affinity of −11.3 kcal/mol (fig. S5, D to F).

Next, we performed a protein-protein interaction analysis between the trimeric S protein and ACE2 using the PatchDock server (*24*). We have also used the FireDock server for further refinements of the complexes. We observed a significant conformational change between the native trimeric S protein RBD-ACE2 complex and the PC786 drug conjugate complex. When the full trimeric S protein RBD binds to ACE2, closed conformation with the highest binding affinity of −40.6 kcal/mol with key residues involved has been observed as depicted by Yan *et al.* (Fig. 5D) (*25*). The closed conformation involves Asp^615^, Thr^376^, Lys^417^, Tyr^453^, Cys^488^, Gly^404^, Tyr^508^, Tyr^421^, and Tyr^453^ of the RBD of S protein and Tyr^613^, Glu^23^, Ala^386^, Ala^384^, Tyr^613^, Ala^614^, Ser^19^, Tyr^20^, Lys^26^, Lys^353^, Gln^388^, Lys^475^, and Arg^559^ of the ACE2 subunit (Fig. 5E). The closed conformation mostly involves hydrophobic residues that play a pivotal role in signal transduction processes/signaling cascade.

When PC786 binds to the RBD of the S protein, upon interaction with ACE2 enzyme, the conformation changed from close to open with the least binding affinity of −6.7 kcal/mol according to the protein-protein interaction analysis that may affect viral attachment and infection (Fig. 5F). The residual changes observed in this complex interaction generally involve Asn^343^, Ala^344^, ^Arg355^, Arg^357^, Ser^399^, Lys^424^, Tyr^453^, Val^512^, Lys^129^, and Ser^155^ of the RBD and N-terminal domain as well. ACE2 enzyme involves Thr^608^, Asp^609^, Trp^606^, Ser^602^, Asn^250^, Glu^238^, Asp^597^, Glu^589^, Ser^602^, Asp^157^, and Asp^136^ in which most of the residues are from the C-terminal domain of ACE2 upon interaction with the drug conjugate complex (Fig. 5G). The analyses mentioned above can be attributed to a predictable conclusion that, when the small-molecule inhibitor binds to the S protein, it substantially affects the binding to the ACE2 domain, which may be helpful to reduce the chances of the signal cascading process in host viral infection. Again, to confirm that we have also considered the FDA-approved drug zanamivir for protein-protein interaction analysis, the analysis revealed that, when zanamivir binds to the RBD-ACE2, the interaction remains in closed conformation with a score of −25.1 kcal/mol, which is more or less similar to the native complex of RBD-ACE2 (fig. S5, G to I). The higher binding affinity reveals that ACE2 strongly binds to the RBD of trimeric S protein that can mediate the cascade of viral replication. Upon binding of antiviral drugs, the binding affinities were less in comparison to the native complex for which PC786 proved to be better from our analyses.

### Immunoinformatics approach for designing T cell and B cell epitopes

**Fig. 6:**
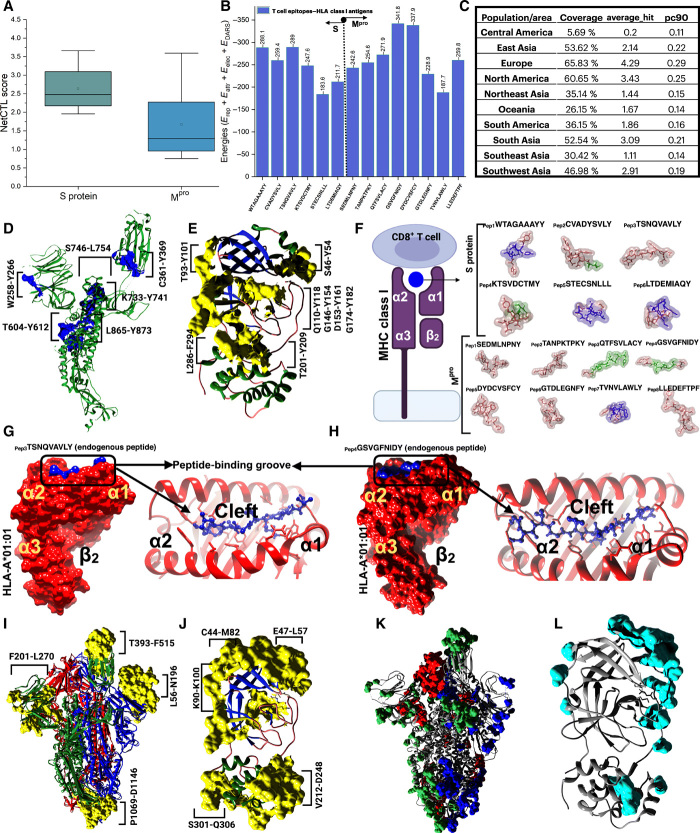
Immunoinformatics approach for finding potential T cell and B cell epitopes. (**A**) Box plot depicting NetCTL scores for predictions of cytotoxic T lymphocyte (CTL) epitopes for rational vaccine design against SARS-CoV-2 S protein (green) and M^pro^ (blue). (**B**) Bar chart plot showing T cell epitope–HLA class I antigens interaction energy scores predicted using ClusPro 2.0 server. (**C**) Combined population coverage analysis of the T cell epitopes predicted from S protein and M^pro^ of SARS-CoV-2. (**D** and **E**) CTL epitopes (blue and yellow colors) identified from S protein (green color, chain A of trimeric S protein) and M^pro^, respectively. (**F**) Schematic of CTL epitopes binding to MHC class I molecules (representing peptide-binding groove present in between two α domains and one β_2_-microglobulin domain. 3D representation of T cell epitopes identified from the PEP-FOLD server for S protein and M^pro^ of SARS-CoV-2, respectively. (**G** and **H**) T cell epitopes, e.g., TSNQVAVLY and GSVGFNIDY of S protein and M^pro^ interaction with HLA-A*01:01 molecules, respectively. The T cell epitopes bind in the cleft (peptide-binding groove) between the two α domains. (**I** and **J**) Identified linear B cell epitopes using BepiPred and ElliPro analyses. The B cell epitopes were represented in yellow color on both trimeric S protein and M^pro^. (**K** and **L**) Predicted discontinuous epitopes (surface presentation) from DiscoTope analysis using trimeric S protein and M^pro^.

In addition to the drug screening approach, integrating immunogenomics using a bioinformatics approach to design various treatments and finding potential candidates in the form of a drug or peptide for SARS-CoV-2 could help during this pandemic outbreak. This immunoinformatics approach could help in designing new vaccines and can overcome the problem of experimentation and time-consuming development process. The discovery of epitope is a key first step in antigen-targeted immunotherapy against viral diseases (*26*). Several studies have proposed strategies to achieve this in the past decade, particularly in the light of antiviral immunotherapy (*27*). The current strategy mainly focuses on identifying B cell epitopes using key structural proteins, i.e., S and M^pro^ of SARS-CoV-2 (*28*). The epitopes identified using the structure-based approach are expected to be most valuable leads. The implied in silico method identifies human leukocyte antigens (HLAs) and T cell epitopes (*29*). That specifies the selection of potent vaccine candidates associated with the transporter associated with antigen processing (TAP) molecules. The designed peptides determined through the immunoinformatics approach served as a foreign substance for the human host cells, thus producing inflammation, demonstrating an allergic reaction. The present study was undertaken to design T cell and B cell epitope–based peptide vaccines against SARS-CoV-2 using the immunoinformatics approach. The approach can serve as a fast-track approach for experimentalists to validate the prediction. The query for SARS-CoV-2 structural and nonstructural proteins resulted in a few pieces of evidence, and thus not much information was obtained. Since the S and M^pro^ proteins were available for SARS-CoV-2, structure-based epitope design was taken into consideration. Both the structural protein, i.e., S protein, and the nonstructural protein, i.e., M^pro^, were antigenic in nature according to VaxiJen 2.0 (*30*). The server predicted both the proteins to be antigenic based on an overall protective antigen prediction score of 0.4512 and 0.4159, respectively, which is beyond the threshold 0.4. The NetCTL 1.2 web server (*31*) was used for both the structures to predict CD8^+^ T cell epitopes based on MHC binding affinity, C-terminal cleavage affinity, TAP transport efficiency, and NetCTL prediction scores (Fig. 6A). The study unraveled six T cell epitopes for the spike protein and eight T cell epitopes for the M^pro^ protein based on a combined score of NetCTL.

The selection is thus chosen to be the best T cell epitopes keeping in mind the MHC binding affinity and can interact with MHC alleles creating an effective immune response. The conservancy of the T cell epitopes is thus necessary to design effective vaccines and can provide immunization effectively. MHC class I immunogenicity score also revealed some significant insights to select the epitopes as high score designates the probability of eliciting an immune response. The selected best T cell epitopes with residue numbering, e.g., WTAGAAAYY, CVADYSVLY, TSNQVAVLY, KTSVDCTMY, STECSNLLL, LTDEMIAQY and SEDMLNPNY, TANPKTPKY, QTFSVLACY, GSVGFNIDY, DYDCVSFCY, GTDLEGNFY, TVNVLAWLY, and LLEDEFTPF, of the spike (Fig. 6D) and M^pro^ (Fig. 6E) proteins respectively were further subjected for MHC class I binding and processing analysis according to IEDB resources. The epitopes were also evaluated for MHC binding affinity, C-terminal cleavage affinity, TAP transport efficiency, proteasome score, and MHC [median inhibitory concentration (IC_50_)] < 200 nm for ensuring high immune response and higher affinity. For the processing of MHC-I, the IEDB analytics method produces an average score for the intrinsic ability of each epitope to be a T cell epitope dependent on the proteasomal synthesis, TAP transport, and MHC-I binding capacity (tables S1 and S2).

Before being introduced to the T cells on the cell’s plasma membrane, the protein is reduced by the cytosolic proteases to tiny peptides in the proteasome, and MHC-I also forms a complex with the peptides. Then, the MHC-I peptide complex is transferred by heat shock proteins and transport-associated proteins to ER. However, the higher the overall score of the epitopes with the HLA alleles assures appearance to the T cell, so it significantly relies on a positive immune response. Eliciting an effective immune response relies not only on a positive identification by HLA molecules with substantial affinity to epitopes but also on the score for antigenicity and immunogenicity. Therefore, the epitopes identified by the vast number of HLA alleles that provide the highest immunogenicity, antigenicity, and safety for humans were flagged as possible epitopes for a robust immune response (tables S1 and S2). Distribution of MHC class I HLA alleles differs across various geographic areas and ethnic groups across the world. Therefore, when planning an efficient vaccine, account must be taken of population coverage. A substantial community density was noticed for the best selected epitopes selected in various geographic regions of the world for both the S and M^pro^ T cell epitopes (Fig. 6C). The S protein T cell epitopes were majorly found to be evident in the European region (fig. S6A). In contrast, M^pro^ T cell epitopes are distributed majorly in the East Asian region (fig. S6B).

The B cell epitope is an appropriate portion of an antigen that is detected in a humoral reaction by either a particular B cell receptor or the elicited antibody (*32*). There are two major groups of B cell epitopes: (i) the B cell epitope continuous and the linear (ii) B cell epitope discontinuous or conformational (*33*–*35*). Most B cell epitopes have been shown to be conformational epitopes, and the amount of this epitope reaches 90%. The detection of antigenic protein B cell epitopes is the main phase in the design of epitope-dependent vaccines, based on B cell epitope prediction methods, e.g., BepiPred and ElliPro, which are linear B cell epitope prediction methods that combinedly predicted 94 linear epitopes using the S protein sequence (Fig. 6I and fig. S6, I and J) and 17 linear epitopes for M^pro^ protein (Fig. 6J and fig. S6, L and M).

DiscoTope analysis gave propensity scores of the individual residues to be evaluated as a discontinuous B cell epitope as depicted in data file S4 and Fig. 6 (K and L). Likewise, other B cell epitope prediction methods such as Chou-Fasman beta-turn prediction, Emini surface accessibility prediction and Kolaskar and Tongaonkar antigenicity also unraveled some B cell epitope regions that could be of a potential interest for researchers in designing new vaccines for SARS-CoV-2. Currently, the vaccinations are mainly based on immunity from B cells. But recently, vaccine based on T cell epitope has been promoted as the host will produce a powerful immune response by CD8^+^ T cell against the infected cell (Fig. 6F). With time, because of antigenic drift, every foreign particle will escape the response of the antibody memory; however, the immune response of T cells also provides long-lasting immunity.

The predictive antigenicity system of Kolaskar and Tongaonkar assesses the antigenicity of a particular epitope. The antigenicity depends on the physiochemical properties of amino acids present in the epitopes. The average antigenic propensity of the protein was 1.037 with a maximum of 1.261 and a minimum of 0.866 in the case of S protein, and an average antigenicity of 1.042 with a minimum 0.844 and a maximum 1.220 was predicted for the M^pro^ protein (fig. S6, E and H), respectively. The Chou-Fasman beta-turn prediction is a method useful for selecting protein regions to be synthesized to produce antipeptide antibodies cross-reacting with the parent protein. As illustrated in fig. S6 (C and F), the predicted B cell epitopes can be experimentally synthesized with a β-turn propensity to produce antipeptide antibodies for both S protein and M^pro^, respectively. The Emini surface accessibility prediction of the B cell epitopes provides evidence that the epitopes found on the surface can be easily accessed by the antibodies and thus elicits an effective immune response. Figure S6 (D and G) represents the epitopes predicted above the threshold value with a maximum propensity of 5.960 and 8.294 in both S and M^pro^ proteins, respectively.

### MHC class I molecules and T cell epitope interaction analysis

In addition to the designing of T cell epitopes, we have predicted the 3D model of the selected epitopes both for S and M^pro^ proteins using the PEP-FOLD (*36*) web-based server for de novo peptide structure prediction to analyze the interactions with particular HLAs, respectively (Fig. 6F). To ensure the binding between HLA molecules and the predicted epitopes, the protein-peptide docking was performed using ClusPro2.0 (*37*) (Fig. 6B). The protein-peptide interactions revealed that T604-Y612 binds to the peptide-binding groove of MHC class I HLA-A*01:01 molecules with an interaction energy of −289 kcal/mol (Fig. 6G). Similarly, the T cell epitope derived from M^pro^, i.e., G146-Y154, also predicted to show high binding affinity (−341.8 kcal/mol) toward MHC class I HLA-A*01:01 molecules (Fig. 6H). On the basis of IEDB MHC-I binding analysis and NetMHCpan, various HLA class I antigens were selected for the receptor molecules on the basis of the availability of the PDB structures. For the T cell epitopes derived from S protein, all the selected T cell epitopes bound to the peptide-binding groove (cleft) of the MHC class I (HLA-A) antigens (fig. S7A). Once a peptide is bound to MHC class I HLA complexes and presented on the cell surface, CD8^+^ T cell epitopes can be detected. Epitope conformation in the HLA-A and HLA-B grooves is of paramount importance not only for epitope affinity and stability but also for epitope interactions. Similar HLA-based epitope interactions were analyzed for the M^pro^ proteins and found evident interactions in the peptide binding groove of MHC class I antigens (fig. S7B). The corresponding epitope HLA class I antigens docking energies have been illustrated in Fig. 6B. The interaction energies obtained for the S- and M^pro^-based epitope interactions with HLA-A and HLA-B suggested that most of the epitopes have good binding affinities toward HLA-A class I molecules. Although, detailed studies based on the interactions have not been explored in this context.

## DISCUSSION

The quick and efficient development of active antiviral agents for therapeutic use is exceptionally challenging because traditional drug development methods usually take years of research and cost billions of dollars. Repurposing approved pharmaceutical drugs and drug applicants provide an alternative approach for rapid identification of potential medication leads and expeditious management of evolving viral infections. In the current study, the combined drug designing and immunoinformatics approach provide a detailed understanding of the vital structural domains involved in either acting as a substrate-binding site or epitope recognition site. Research teams in companies and universities are currently developing more than 90 vaccines against SARS-CoV-2 (*38*). At least eight types of coronavirus are being tested, relying on different viruses or viral parts (*38*). The structure-based immunoinformatics approach may help identify vital structural domains and active sites that can provide a basis for development of protein-based vaccines targeting mainly spike glycoprotein of SARS-CoV-2. We do acknowledge several limitations in validating our in silico proposed work due to the lack of experimental support. However, the strategies mentioned above can reduce the resources and expenses for researchers involved directly with the experimental and clinical studies, with a higher probability of obtaining the desired responses and fewer trials and recurrences of mistake. Our strategy will reduce the translational distance between preclinical test results and clinical outcomes, and thus help to address a major challenge for the rapid development of practical treatment approaches for the ongoing SARS-CoV-2 pandemic.

## MATERIALS AND METHODS

### Protein structure retrieval

We have retrieved the crystal structures of prefusion SARS-CoV-2 spike glycoprotein with an RBD (S) (PDB ID: 6VSB) and the M^pro^ in complex with an inhibitor N3 (PDB-ID: 6 LU7) from PDB. For the complex interaction analyses with RBD of S protein, we have taken the structure of native human ACE–related carboxypeptidase (ACE2) (PDB ID: 1R42). We have used UCSF Chimera and Discovery Studio Visualizer to visualize and analyze the interactions.

### Virtual screening and molecular docking

The antiviral drug compounds were retrieved from the ChEMBL database with a search query term “antiviral drugs” and “coronavirus” that resulted in 640 chemical compounds with the corresponding filters for data availability, e.g., SMILES and Structure Data File formats. We have screened the antiviral drug molecules for RO5 violations and refined them to get 3D coordinates using the Open Babel command-line tool. All the structures that have passed the RO5 rule have been subjected for further refinement using MarvinSketch. The drug compounds and the corresponding target receptors, e.g., 6VSB and 6 LU7, were submitted for virtual screening using AutoDock Vina. The virtually screened best compounds were then docked with the target receptors again to ensure the conformation poses and binding affinities. We have performed blind docking as the location of binding site is unknown for both target receptors. The grid for the target receptors was set to 126 Å by 126 Å by 126 Å with a spacing of 1.000 Å. The interactions were visualized using Discovery Studio Visualizer. Furthermore, we have again docked the best-screened compounds to the complex of RBD-ACE2 in two conformations: (i) partial chain A–RBD of S protein with ACE2 complex and (ii) full trimeric S protein with ACE2 complex using AutoDock Vina.

### MD simulation

MD simulation using GROMACS v.2019.2 has been performed for the complex molecules (drug bound proteins). We obtained the topologies for all the small antiviral molecules from the PRODRG database. We have optimized the parameters of the target receptor and the drug molecules using the GROMOS96 54a7 force field. The complex systems were placed in a periodic cubic box solvated with simple point charge solvent molecules. Periodic boundary conditions with a 15-Å cutoff for nonbonded interactions were applied, with the particle mesh Ewald method applied to account for the long-range electrostatic interactions. The system was neutralized with Na^+^ counterions to attain equilibration. Energy minimization and equilibration were carried out in three steps as follows: (i) We minimize the whole system containing ions, solvent, protein, and ligand for up to 50,000 steps using a steepest-descent algorithm. (ii) Constraints were added to protein and the ligand dimer for 100 ps during heating using NVT (number of atoms, volume, temperature) ensemble with leapfrog integrator and linear constraint solver holonomic constrains. (iii) NPT ensemble was used at constant pressure (1 bar) and temperature (300 K) for 100 ps using a time step of 2 fs for equilibration phase 2. The SHAKE algorithm was used to constraint hydrogen to heavy atom bonds. The MD production phase for all the systems has been simulated for 10 ns with a time step of 2 fs. Furthermore, after 10-ns simulation, the protein-ligand interaction energy was evaluated to compute the nonbonded interaction energy and short-range nonbonded energies, which were quantitatively reproduced with energy profiles generated by GROMACS tools. Furthermore, we used MM-PBSA to calculate the polar and nonpolar solvation energies with corresponding binding energy decomposition of the complexes. MM-PBSA calculates the free energy of the docked complex (the binding free energy of the protein with ligand in a solvent medium) where the general expression of the term can be depicted as

$$\Delta G_{\text{binding}}=G_{\text{complex}}-(G_{\text{protein}}+G_{\text{ligand}})$$ (1)

where *G*_complex_ is the total free energy of the protein-ligand complex and *G*_protein_ and *G*_ligand_ are total free energies of the isolated protein and ligand in solvent, respectively.

$$G_{x}=\langle E_{\text{MM}}\rangle -\mathrm{TS}+\langle G_{\text{solvation}}\rangle$$ (2)

where *x* is the protein or ligand or protein-ligand complex. ⟨*E*_MM_⟩ is the average molecular mechanics potential energy in a vacuum. *TS* refers to the entropic contribution to the free energy in a vacuum where *T* and *S* denote the temperature and entropy, respectively. The l term ⟨*G*_solvation_⟩ is the free energy of solvation.

$$E=E_{\text{bonded}}+E_{\text{nonbonded}}=E_{\text{bonded}}+(\;E_{\text{vdW}}+E_{\text{elec}})$$ (3)

where *E*_bonded_ is bonded interactions consisting of bond, angle, dihedral, and improper interactions. The nonbonded interactions (*E*_nonbonded_) include both electrostatic (*E*_elec_) and van der Waals (*E*_vdW_) interactions depicted using a Coulomb and Lennard-Jones potential function, respectively.

Moreover, the free energy of solvation, which is the energy required to transfer a solute from a vacuum into the solvent, has been calculated including polar and nonpolar solvation energies that can be depicted as

$$G_{\text{solvation}}=G_{\text{polar}}+G_{\text{nonpolar}}$$ (4)

where *G*_polar_ and *G*_nonpolar_ are the electrostatic and nonelectrostatic contributions to the solvation free energy, respectively.

### Protein-protein interaction

To predict the conformational changes upon binding of ACE2 to the trimeric S protein RBD, we have used PatchDock and FireDock for protein-protein interaction analysis. UCSF Chimera was used for the post protein-protein interaction analyses.

### Antigenicity and T cell epitope identification

We have retrieved the protein FASTA sequence of SARS-CoV-2 isolate Wuhan-Hu-1, complete genome sequence bearing ID NC_045512.2 for the epitope screening. The prediction of protective antigens and subunit vaccines was evaluated using VaxiJen 2.0 with default parameters. The NETCTL 1.2 server was used for the T cell epitope identification. The method integrates MHC-I binding, proteasomal C-terminal cleavage, TAP transport, and combinatorial scores for the prediction of epitopes. For MHC-I binding, IEDB tools have been used to get the best selected epitopes based on the stabilized matrix base method and inhibitory concentrations (IC_50_) values for peptide binding to MHC-class I molecules. Furthermore, the selected epitopes were further processed to obtain the specificity to TAP transport, proteasomal cleavage, TAP transport, and MHC-I. The web-based tool IEDB has been used for population coverage analysis.

### Immunogenicity prediction

The IEDB MHC class I immunogenicity tool and the European Molecular Biology Open Software Suite (EMBOSS) were used for immunogenicity prediction. The algorithm prediction was based on immunogenicity and antigenic scores.

### T cell epitope structure prediction

The selected T cell epitopes were subjected to the PEP-FOLD server to predict the 3D structure to be able to perform the protein-peptide interaction with HLA-A and HLA-B class I molecules.

### B cell epitope prediction

IEDB resources were used to classify B cell antigenicity such as Kolaskar and Tongaonkar antigenicity scale, Emini surface usability prediction, Karplus and Schulz versatility prediction, and BepiPred linear epitope prediction analysis. The Chou-Fasman beta-turn prediction tool is used as the antigenic sections of a protein belong to the β-turn areas. We have also used ElliPro and DiscoTope to predict linear and discontinuous peptides, respectively.

### Molecular interaction of epitopes to HLA class I molecules

The T cell epitopes were further processed for interaction analysis using HLA class I molecules using ClusPro 2.0. ClusPro 2.0 was based on ranking models by cluster size where the ligands were rotated 70,000 conformations. The server also predicts the cluster size and interacting members and gives best models with the lowest energies.

### Statistical analysis

All statistical data analyses were performed in Origin 2018. Linear curve fitting has been performed using independent and dependent variables with the goal of defining a “best fit” model of the relationship. Use of weighted least-square method to fit a linear model function to specified data has been performed. Box plots, scatter plots, and bar graphs have been depicted to represent the data.

## Supplementary Material

abb8097_Data_file_S4.xlsx

abb8097_SM.pdf

abb8097_Data_file_S2.xlsx

abb8097_Data_file_S1.xlsx

abb8097_Data_file_S3.xlsx

## SUPPLEMENTARY MATERIALS

Supplementary material for this article is available at http://advances.sciencemag.org/cgi/content/full/6/28/eabb8097/DC1
