# Supplementary material for: Structure-based drug designing and immunoinformatics approach for SARS-CoV-2
Source: Sci Adv. 2020 Jul 10;6(28):eabb8097. doi: 10.1126/sciadv.abb8097 (PMC7319274; doi:10.1126/sciadv.abb8097)
Supplement: abb8097_SM.pdf [file abb8097_SM.pdf]

Science Advances
AAAS

advances.sciencemag.org/cgi/content/full/sciadv.abb8097/DC1

Supplementary Materials for

Structure-based drug designing and immunoinformatics approach for SARS-CoV-2

Pritam Kumar Panda*, Murugan Natarajan Arul, Paritosh Patel, Suresh K Verma, Wei Luo, Horst-Günter Rubahn, Yogendra Kumar Mishra, Mrutyunjay Suar, Rajeev Ahuja*

*Corresponding author. Email: rajeev.ahuja@physics.uu.se; pritam.panda@physics.uu.se

Published 24 June 2020, *Sci. Adv.* **6**, eabb8097 (2020)
DOI: 10.1126/sciadv.abb8097

This PDF file includes:

Figs. S1 to S7
Tables S1 to S2

Other Supplementary Material for this manuscript includes the following:

(available at advances.sciencemag.org/cgi/content/full/sciadv.abb8097/DC1)

Datafiles S1-S4

Supplementary Figures

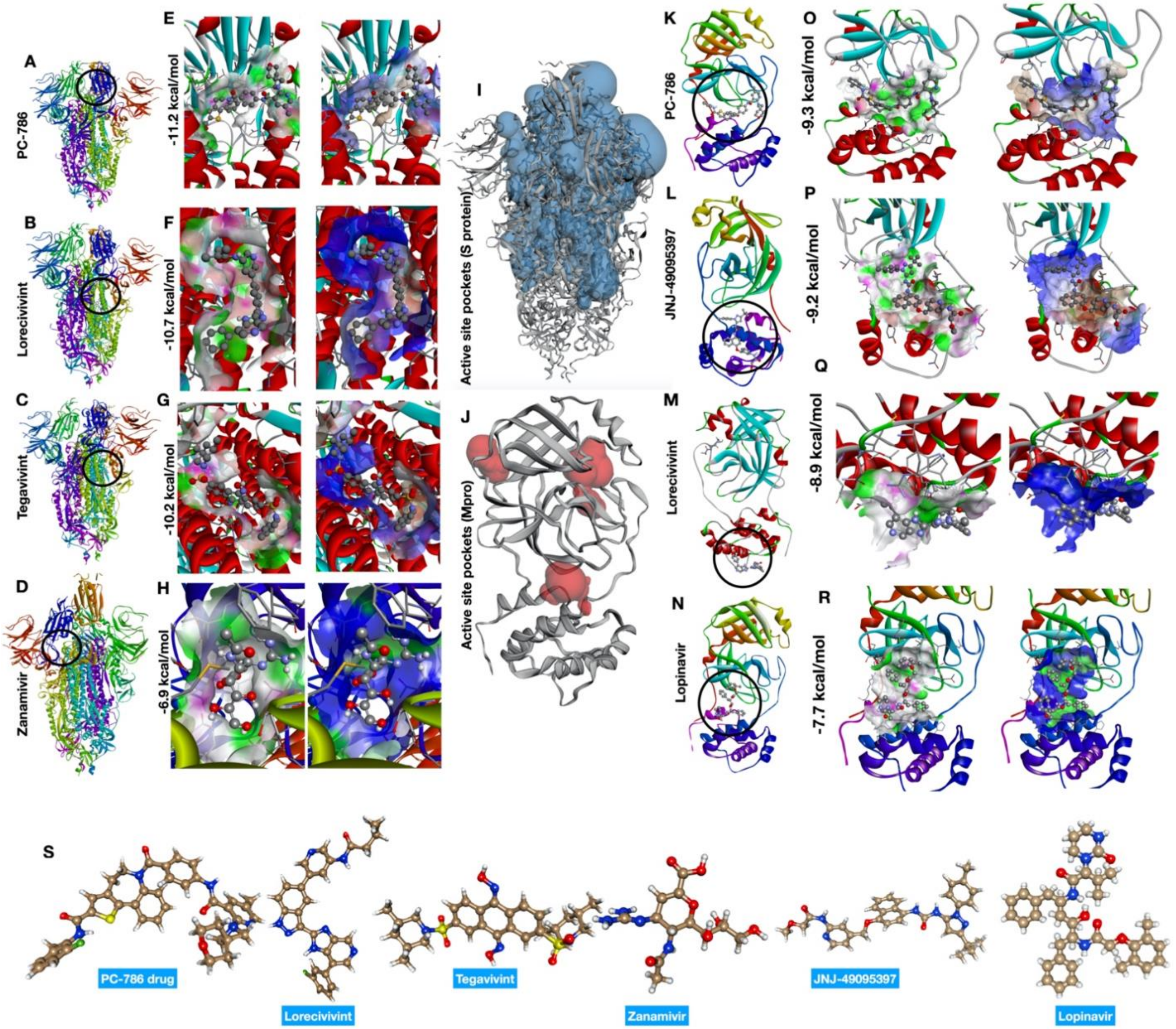

Fig S1: Molecular docking analyses of antiviral compounds against S and M_{pro} proteins of SARS-CoV-2 (A-D) Molecular docking interaction of antiviral drugs to the trimeric S protein. **(E-H).** Close view of the binding modes of antiviral drugs to the S protein with hydrogen bonding and solvent accessible surface area. **(I-J)** Active site pockets of the trimeric S protein (blue) and M_{pro} (red) analyzed using CastP. **(K-N).** Binding modes of antiviral drugs to the M_{pro}. **(O-R)** Close view of the binding modes of antiviral drugs to the M_{pro} with hydrogen bonding and solvent accessible surface area. **(S)** 3D structure of the best screened and FDA approved antiviral compounds.

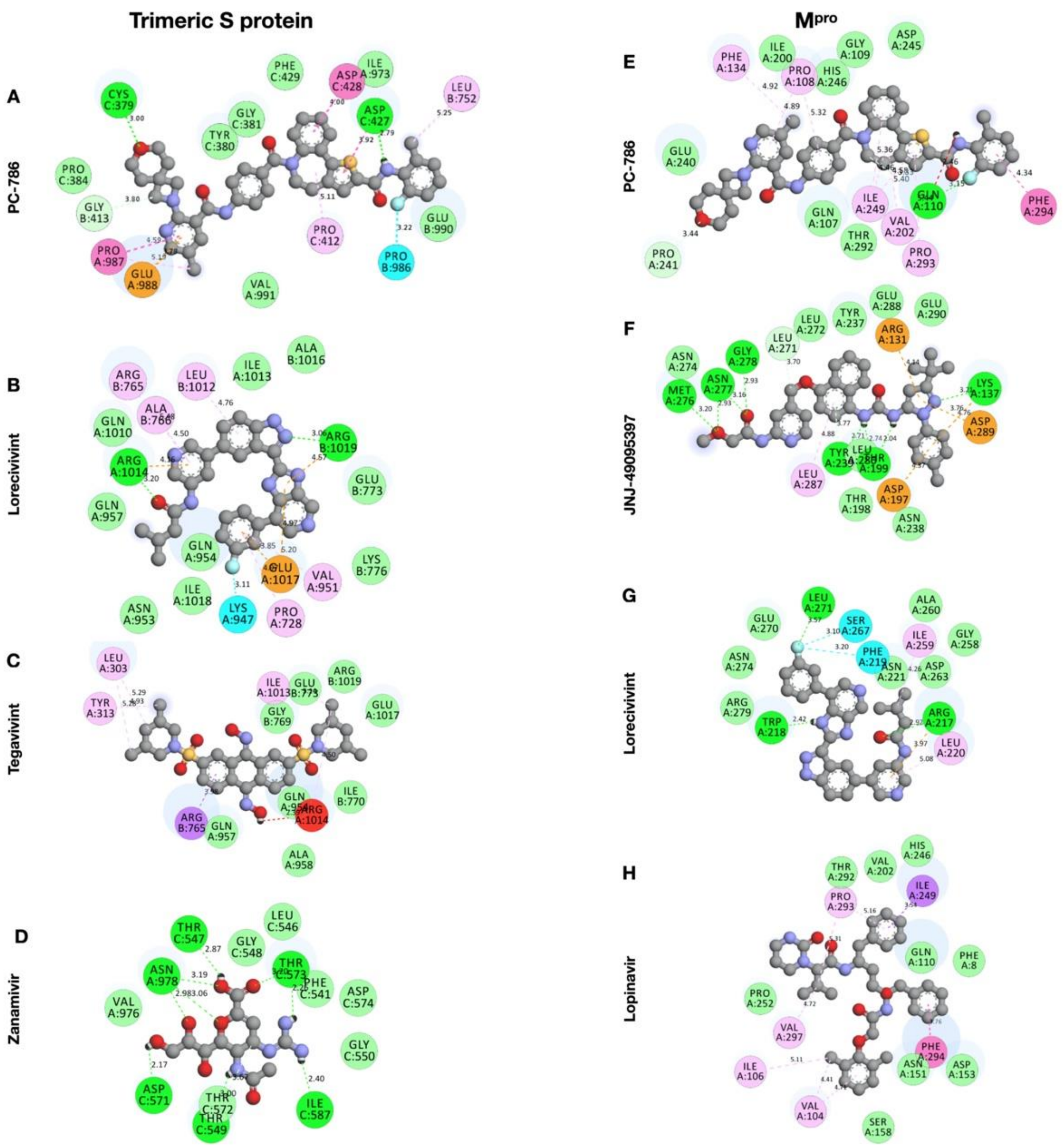

Fig S2: 2D interaction plot of the antiviral drug compounds bound to trimeric S protein and M$_{pro}$. (A-D) Interaction of best-selected compounds with one FDA approved drug Zanamivir for trimeric S protein. **(E-H)** 2D representation of antiviral drugs with one FDA approved drug Lopinavir for M$_{pro}$.

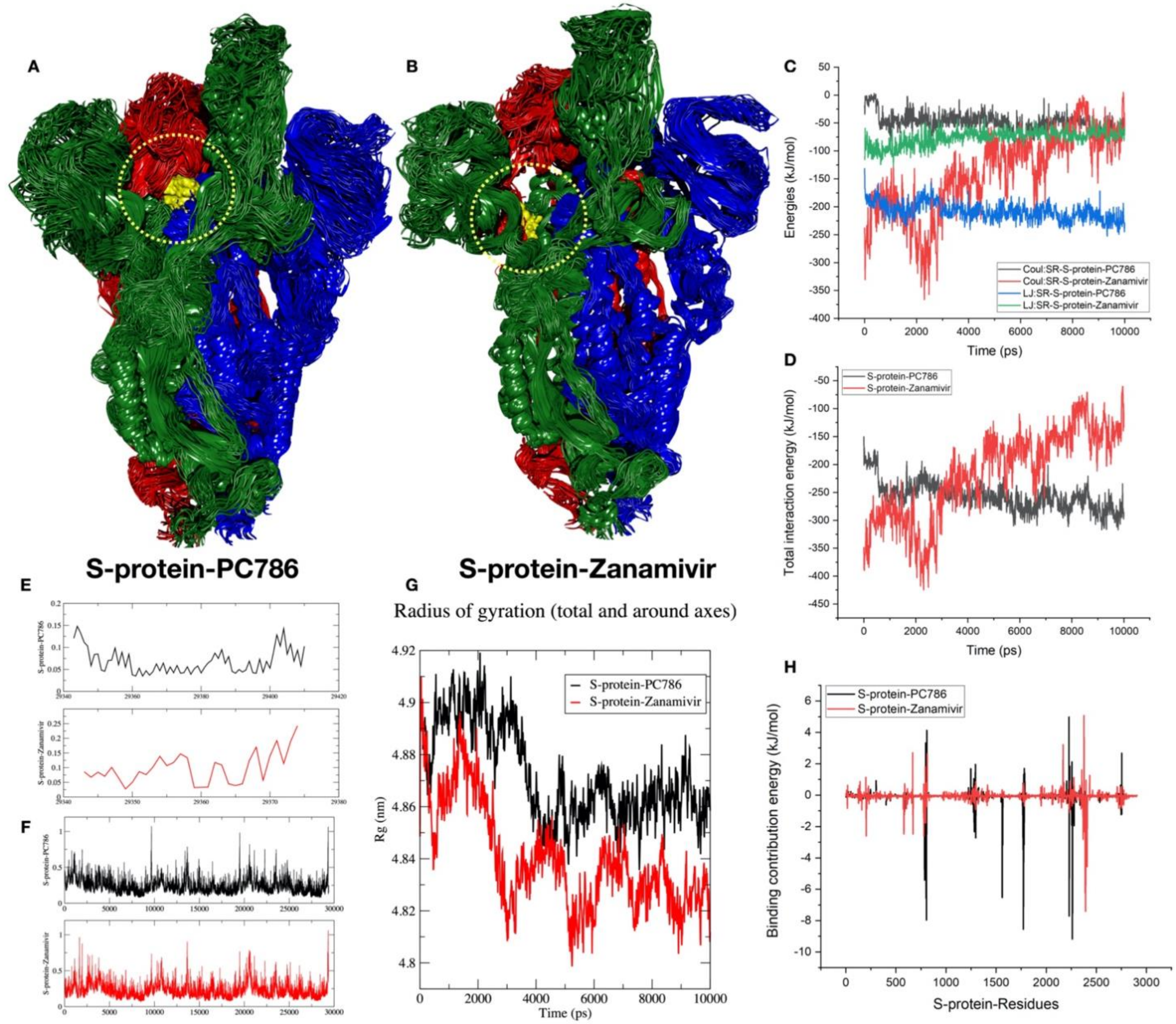

Fig S3: Molecular dynamics simulation of trimeric S protein bound to antiviral drugs (A, B) Cluster of 10ns simulated trimeric S protein conjugated with PC786 and Zanamivir antiviral compounds. **(C, D)** Energies are corresponding to short-range Coulombic interaction energy (kJ/mol), Lennard-Jones energy (kJ/mol), and total interaction energies (kJ/mol). **(E, F)** Root Mean Square Fluctuation (RMSF) analysis of antiviral drug and the complex during 10ns simulation. **(G)** The radius of gyration depicting the compactness of the protein during the 10ns simulation. **(H)** The energy contribution of residues towards binding to the drug calculated using MM-PBSA during the 10ns simulation.

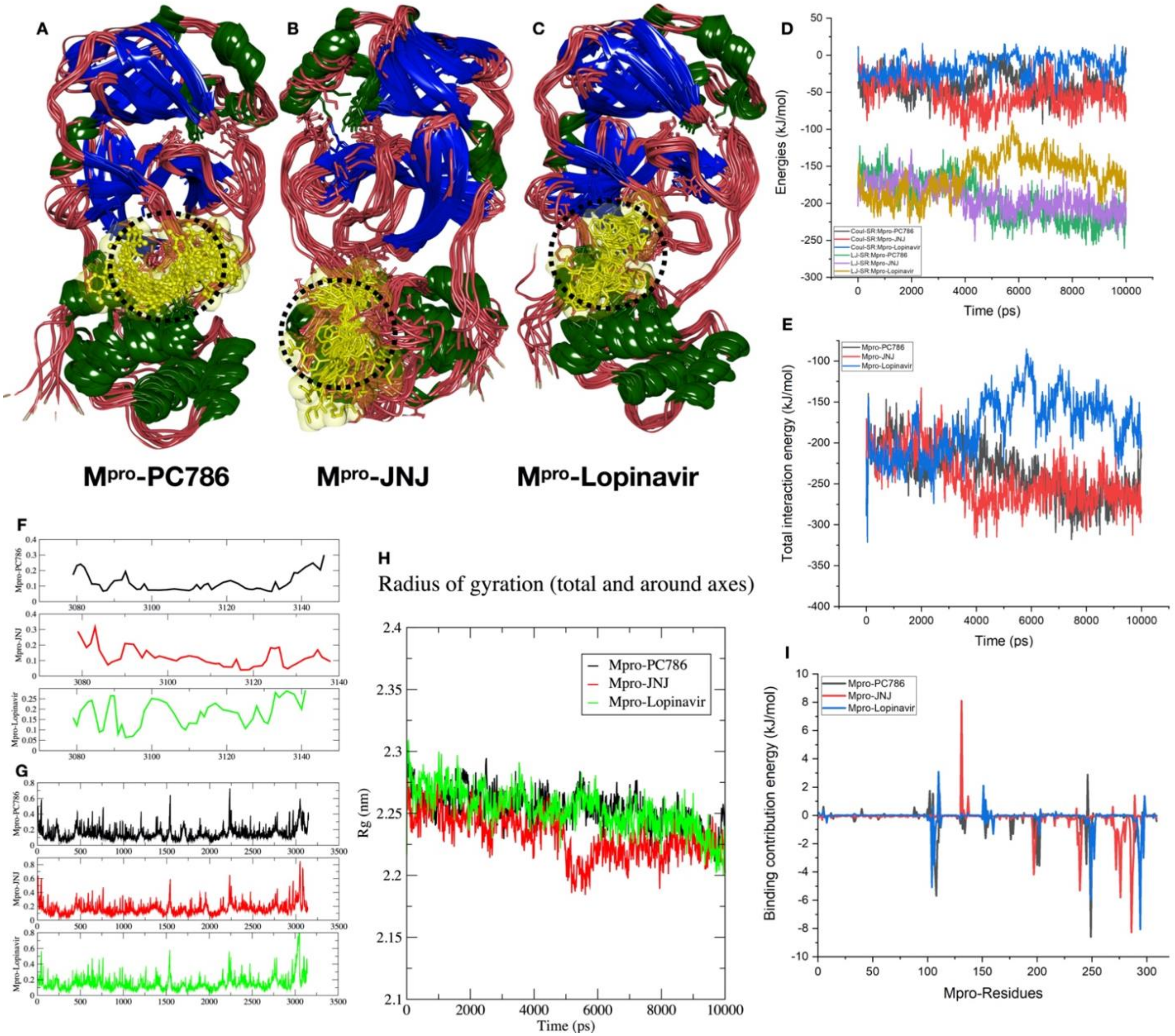

Fig S4: Molecular dynamics simulation of M_{pro} protein bound to antiviral drugs (A, B, C) Cluster of 10ns simulated M_{pro} conjugated with PC786, JNJ, and Lopinavir antiviral compounds. **(D, E)** Energies are corresponding to short-range Coulombic interaction energy (kJ/mol), Lennard-Jones energy (kJ/mol), and total interaction energies (kJ/mol). **(F, G)** Root Mean Square Fluctuation (RMSF) analysis of antiviral drug and the complex during 10ns simulation. **(H)** The radius of gyration depicting the compactness of the protein during the 10ns simulation. **(I)** The energy contribution of residues towards binding to the drug calculated using MM-PBSA during the 10ns simulation.

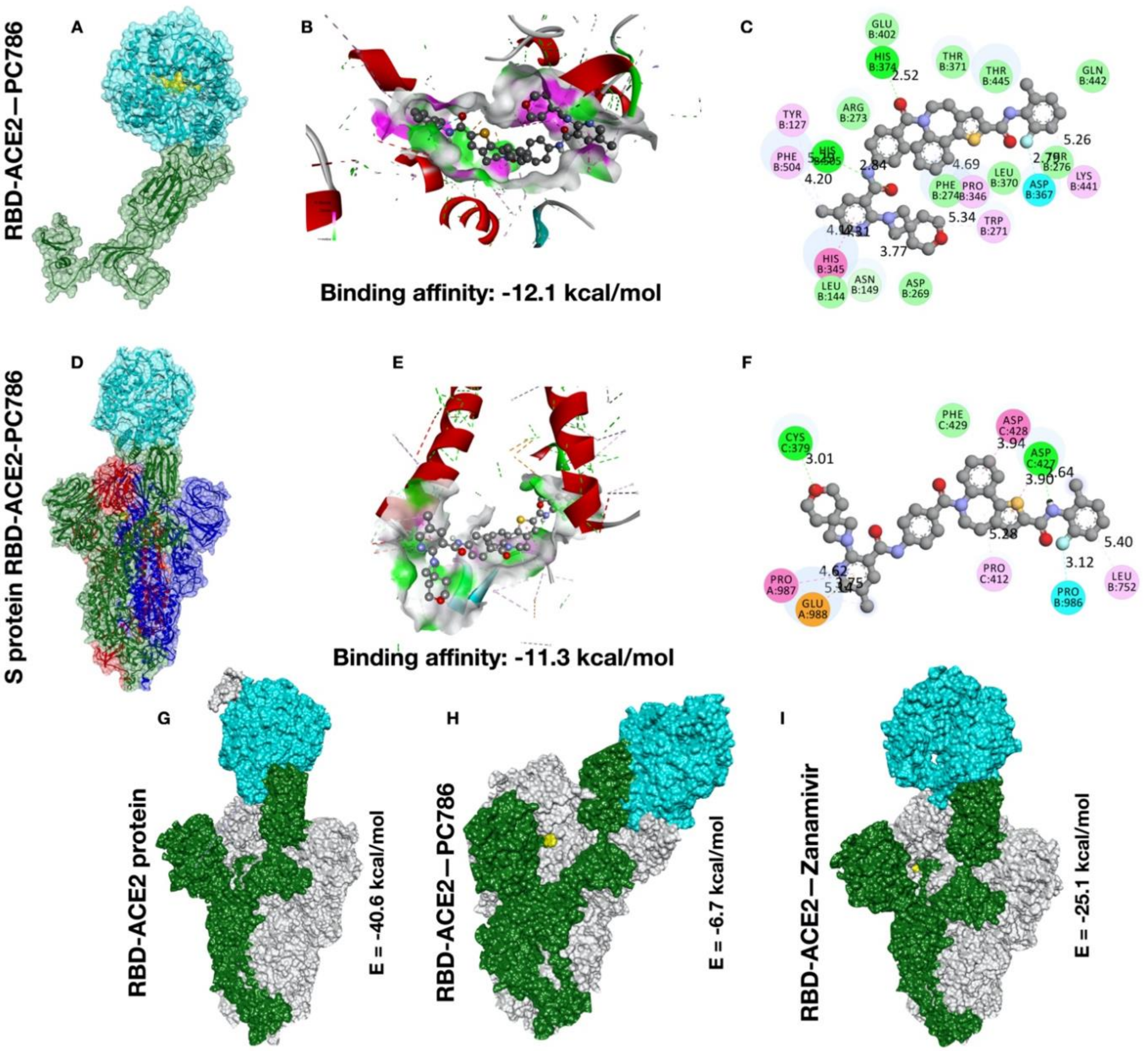

Fig S5: Inhibition of S protein RBD domain complexed with ACE2 (A) Complex of S protein RBD-ACE2 bound to PC786. **(B)** Close view of the interaction of PC786 to the complex. **(C)** 2D representation of the interaction involving key residues. (D) Full complex of trimeric S protein RBD domain-ACE2 conjugated with PC786 drug. **(E)** Close view of the interaction of PC786 to the complex. **(F)** 2D representation of the interaction involving key residues. **(G)** Trimeric S protein RBD domain binding to ACE2 in a closed conformation **(H)** Open conformation of PC786 drug conjugate full RBD-ACE2 complex. **(I)** Closed conformation of Zanamivir drug conjugate with full RBD-ACE2 complex.

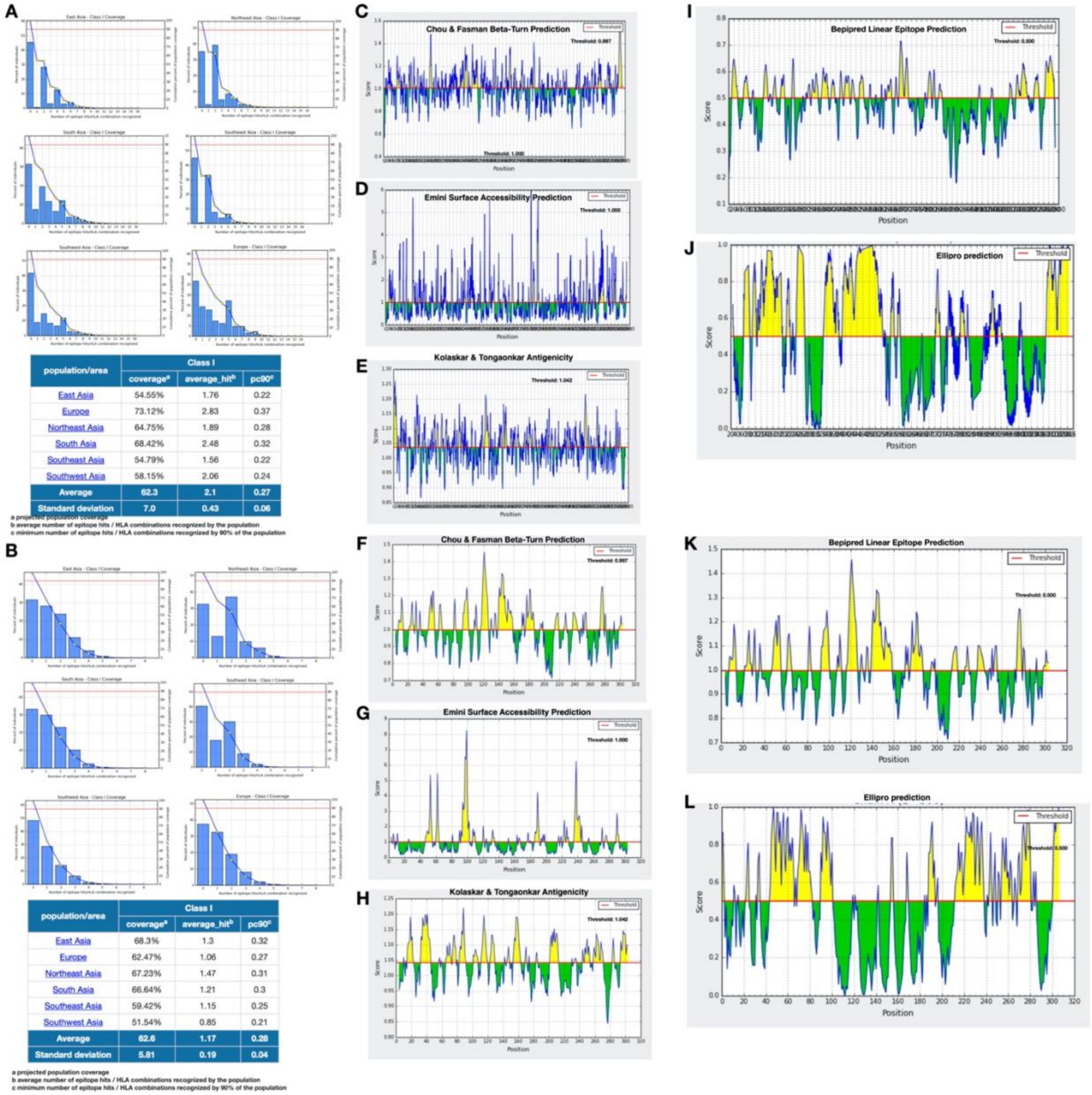

Fig S6: Immunoinformatics approach for finding T cell and B cell epitopes from S protein and M_{pro} of SARS-CoV-2, respectively. (A, B) Population coverage analysis of the T cell epitopes predicted from S protein and M_{pro} of SARS-CoV-2, respectively, with the percentage of coverage and average hits of the T cell epitopes across the continents. Linear B cell epitope sequence properties i.e., **(C and F)** Chou & Fasman beta-turn prediction analysis of B cell epitopes **(D and G)** Emini surface accessibility prediction **(E and H)** Kolaskar & Tongaonkar antigenicity prediction for S protein and M_{pro}, respectively. **(I and K)** B cell linear epitope prediction using BepiPred server from IEDB tools for S and M_{pro}, respectively. **(J and L)** Ellipro prediction for S and M_{pro} respectively.

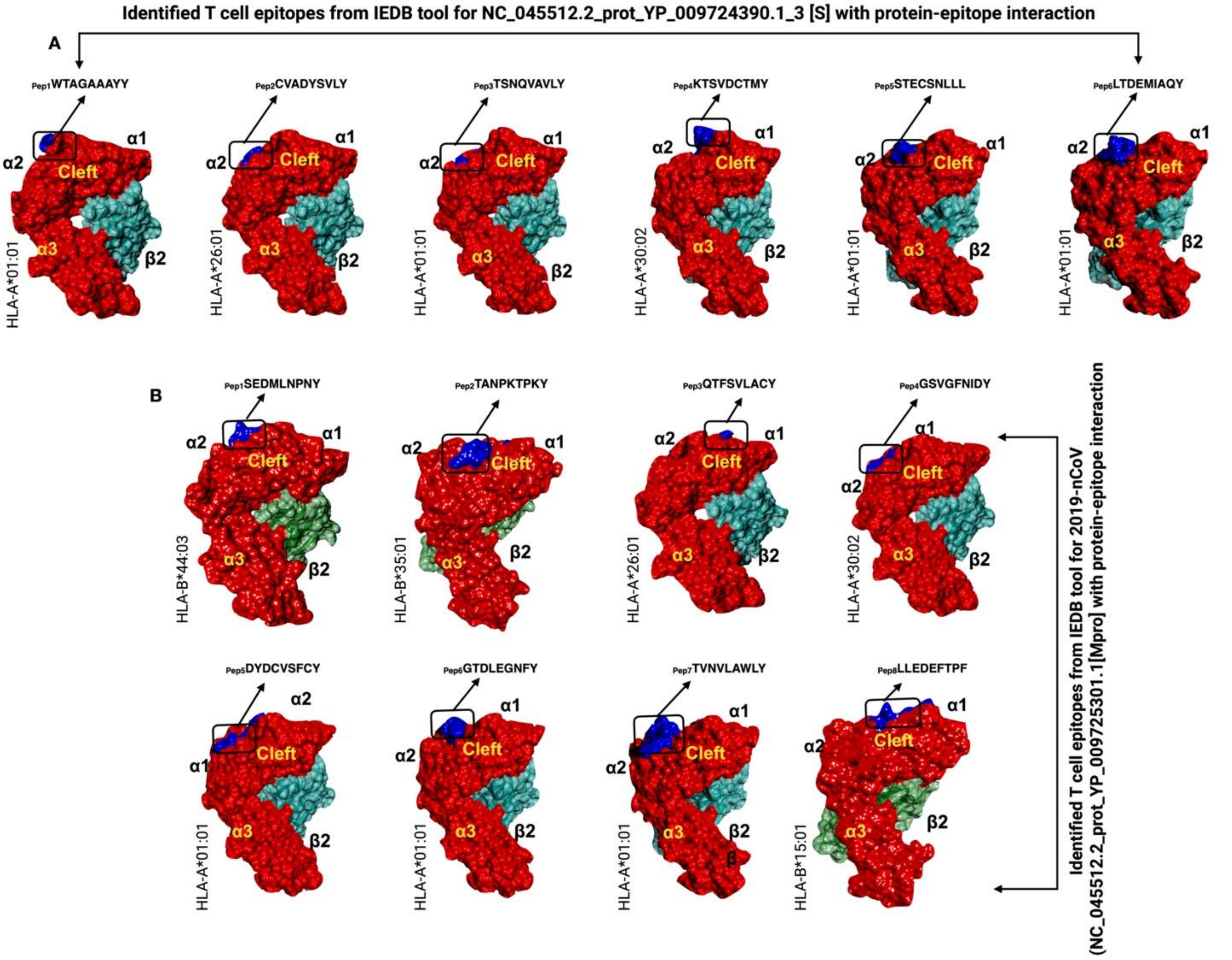

Fig S7: T cell epitope interaction with MHC class I HLA alleles (A, B) Predicted T cell epitopes (blue surface) from S protein (Pep1-Pep6) (top) and M_{pro} (Pep1-Pep8) (below) interaction with different HLA-A class I antigens (red surface) using ClusPro 2.0 respectively. The T cell epitopes bind in the cleft (peptide-binding groove) between the two α domains and β microglobulin.

Table S1: Identified T cell epitopes from IEDB tool for NC_045512.2_prot_YP_009724390.1_3 [S] protein with protein-epitope interaction energies.

No	Antigenic Peptides	Cluspro Lowest Energies		Allelic specificity	MHC Class I Immunogenicity Score	NETMHC Plan Binding level	Antigenic Peptide selectivity	MHC Binding Affinity	C-Terminal Cleavage Affinity	TAP transport efficiency	NETCTL Prediction Score	MHC I Processing			
		HLA-A specific	HLA-B specific									Proteasome	Tap score	MHC score	MHC (IC50)
1	WTAGAAAYY	-288.1	-	HLA-B*15:01<br>HLA-B*35:01<br>HLA-A*68:01<br>HLA-A*30:02<br>HLA-A*26:01<br>HLA-A*01:01	0.15259	Strongly binds	NETCTL, MHC Binding-I prediction	0.7953	0.9723	2.779	3.6616	1.24	1.24	-1.06	11.6
2	CVADYSVLY	-259.4	-	HLA-B*35:01<br>HLA-A*68:01<br>HLA-A*30:02<br>HLA-A*26:01	0.02757	Strongly binds	NETCTL, MHC Binding-I prediction	0.6735	0.7339	2.779	3.1128	1.51	1.38	-1.39	24.4
3	TSNQVAVLY	-289	-	HLA-B*57:01<br>HLA-A*68:01<br>HLA-B*35:01<br>HLA-B*58:01<br>HLA-A*30:02<br>HLA-A*01:01	-0.01327	Strongly binds	NETCTL, MHC Binding-I prediction, NET MHC Plan	0.6559	0.944	2.991	3.0758	1.47	1.3	-1.83	68.1
4	KTSVDCTMY	-247.6	-	HLA-A*01:01<br>HLA-A*30:02	-0.09595	Strongly binds	NETCTL	0.5348	0.9764	3.18	2.5759	1.26	1.31	-1.61	41.1
5	STECSNLLL	-183.6	-	HLA-A*01:01	-0.11115	Weakly binds	NETCTL, EMBOSS	0.4908	0.9649	3.016	2.3795	1.34	1.16	-1.61	40.3
6	LTDEMIAQY	-211.7	-	HLA-B*58:01<br>HLA-B*35:01<br>HLA-A*30:02<br>HLA-A*01:01	-0.20478	Weakly binds	NETCTL, MHC Binding-I prediction	0.5136	0.8879	0.703	2.3492	1.21	1.21	-0.72	5.2

Table S2: Identified T cell epitopes from IEDB tool for 2019-nCoV (NC_045512.2_prot_YP_009725301.1[M_{pro}] protein with protein-epitope interaction energies.

No	Antigenic Peptides	Cluspro Lowest Energies		Allelic specificity MHC processing and NETMHC Plan	MHC Class I Immunogenicity Score	NETMHC Plan Binding level	Antigenic Peptide selectivity	MHC Binding Affinity	C-Terminal Cleavage Affinity	TAP transport efficiency	NETCTL Prediction Score	MHC I Processing			
		HLA-A specific	HLA-B specific									Proteasome	Tap score	MHC score	MHC (IC50)
1	SEDMLNPNY		-242.6	HLA-B*44:02<br>HLA-B*44:03	-0.19953	Weakly binds	NETCTL, MHC Binding-I prediction	0.1528	0.8406	2.676	0.9088	1.54	1.16	-2.18	151.1
2	TANPKTPKY		-254.6	HLA-B*53:01<br>HLA-B*15:01<br>HLA-B*58:01<br>HLA-A*01:01<br>HLA-A*30:02<br>HLA-B*35:01	-0.32208	Weakly binds	NETCTL, MHC Binding-I prediction	0.1676	0.9755	2.723	0.9942	1.41	1.18	-2.04	110.8
3	QTFSVLACY	-271.9		HLA-A*68:01<br>HLA-A*30:02<br>HLA-A*26:01	-0.09719	Weakly binds	NETCTL, MHC Binding-I prediction, NET MHC Plan	0.2625	0.9725	2.998	1.4104	1.4	1.3	-2.22	165.2
4	GSVGFNIDY	-341.8		HLA-A*30:02	0.28089	Strongly binds	NETCTL	0.3112	0.9565	2.857	1.6075	-	-	-	-
5	DYDCVSFCY	-337.9		HLA-A*01:01	-0.09528	Strongly binds	NETCTL, EMBOSS	0.2097	0.9722	2.706	1.1717	-	-	-	-
6	GTDLEGNFY	-228.9		HLA-A*30:02<br>HLA-A*01:01	0.18838	Strongly binds	NETCTL, MHC Binding-I prediction, NET MHC Plan	0.793	0.6229	2.702	3.5954	1.42	1.17	-0.99	9.7
7	TVNVLAWLY	-187.7		HLA-A*01:01	0.24595	Strongly binds	NETCTL, MHC Binding-I prediction, NET MHC Plan, EMBOSS	0.6255	0.8852	2.957	2.9365	1.43	1.28	-2.22	164.7
8	LLEDEFTPF		-259.8	HLA-B*15:01	0.2888	Strongly binds	NETCTL, MHC Binding-I prediction, NET MHC Plan	0.1132	0.9503	2.568	0.7517	0.94	1.11	-2.09	121.8

Supplementary data files:

Supplementary data file S1: List of 640 small molecule antiviral compounds obtained from the ChEMBL database with molecular descriptors. Binding affinity scores for both S protein and M_{pro}. Best selected compounds from virtual screening of antiviral compounds with S protein and main protease with Autodock Vina scores. Description of the best-selected compounds (potent inhibitors for SARS-CoV-2) with clinical features, i.e., Treatments, clinical phase, and molecular descriptors. Binding affinities of clinically approved FDA antiviral drugs. ACE2-RBD complex binding affinity scores.

Supplementary data file S2: Key residues involved in active site pockets of S protein (PDB-ID 6VSB) and M_{pro} (PDB-ID 6LU7) obtained from the CastP server.

Supplementary data file S3: MMPBSA analysis for M_{pro}-PC786, M_{pro}-JNJ, M_{pro}-Lopinavir, S protein-PC786, S-protein-Zanamivir with binding free energies and residual contributions.

Supplementary data file S4: B cell epitope identification using BepiPred, Ellipro, and Discotope for S protein and main protease. Supplementary Table 1 (S1) Identified T cell epitopes from IEDB tool for NC_045512.2_prot_YP_009724390.1_3 [S] protein with protein-epitope interaction energies (S2) Identified T cell epitopes from IEDB tool for SARS-CoV-2 (NC_045512.2_prot_YP_009725301.1[M_{pro}] protein with protein-epitope interaction energies.
